# Validation of a functional screening instrument for dementia in an elderly sri lankan population: comparison of modified bristol and blessed activities of daily living scales

**DOI:** 10.1186/1756-0500-3-268

**Published:** 2010-10-26

**Authors:** S Umayal, M Kulathunga, S Somaratne, S Srikanth, S Kathriarachchi, Ranil De Silva

**Affiliations:** 1 Dept of Anatomy, Faculty of Medical Sciences, University of Sri Jayewardenepura, Nugegoda, Sri Lanka; 2 Dept of Psychiatry, Faculty of Medical Sciences, University of Sri Jayewardenepura, Nugegoda, Sri Lanka; 3 Dept of Botany, Open University of Sri Lanka, Nawala, Sri Lanka; 4 Neurology unit, Apollo Hospital, Colombo-05, Sri Lanka

## Abstract

**Background:**

Cognitive tests have been used in population surveys as first stage screens for dementia but are biased by education. However functional ability scales are less biased by education than the cognitive scale and thus can be used in screening for dementia.

**Objective:**

To validate Activities of Daily Living (ADL) scale appropriate for use in assessing the presence of dementia in an elderly population living in care homes in Sri Lanka.

**Method:**

Sinhalese version of the modified Bristol and Blessed scale was administered to subjects aged 55 years and above residing in 14 randomly selected elders' homes. Receiver Operating Characteristic (ROC) was used to determine the cut-off scores of both the scales.

**Results:**

Based on the ROC analysis, optimal cut off score of the modified Bristol scale was 20 with a sensitivity of 100%, specificity of 74.2% and the area under the curve 0.933(95% CI: 0.871-0.995) while the optimal cut off score of the modified Blessed scale was 10.5 with a sensitivity of 100%, specificity of 71% and the area under the curve 0.892 (95% CI: 0.816-0.967).

**Conclusion:**

The findings confirm that both the scales can be used in screening for dementia in the elderly living in care homes in Sri Lanka.

## Background

Dementia is defined as cognitive decline of sufficient magnitude to impair day to day functioning. Cognitive tests (e.g. Mini Mental State Examination -MMSE) [1] have been used in population surveys as first stage screens for dementia [2-4] but are biased by education [5,6] and hence it is more difficult to use them as case finding tools in primary care especially in low literacy settings [7]. However functional ability scales are less biased by education than the cognitive scales [8] thus can be used in screening for dementia in conjunction with cognitive screening instrument [9].

Sri Lanka's population has a literacy rate of 91%, higher than that expected for a third world country and it has the highest literacy rate in South Asia [10]. However, the studies from elder's homes in Western province of Sri Lanka has confirmed that majority of the elders resides in elders homes are illiterate [6]. Administration of cognitive tests to above setting may be demeaning to a patient and requires a tool that would adequately detect dementia in early stage.

In the last decade the elderly population living in developing countries has increased by 200-280% compared with a mere 30-40% increase in the developed nations [11]. Of the world's 580 million elderly (>60 yrs), 335 million (61%) live in developing countries [12]. A World Bank report on Sri Lanka's aging population has revealed that the country is rated as the fastest aging population in South Asia. Sri Lanka's share of population over 60 years old in 2000 was 9.2% which will reach almost 30% by 2050 [13]. (The population of the Colombo city was approximately 2,251,274 [14]). The declining birth rate, coupled with the high life expectancy of 71.7 years for males and 76.4 years for females [15] and the protracted war has led to the increase in the aged population [16].

In Sri Lanka, a developing country, rapidly aging population has led to the decrease in traditional family support and increase in institutionalized for elderly people. Reflecting this change in the age distribution, there has been an increase in the number of public care homes for elders under the Department of Social Services in Sri Lanka from a total of 68 homes in 1987 to 162 homes in 2003 (this number excluding paying homes) [17].

Standardized functional ability tests are commonly used in Western countries [7,18,19] and in few developing countries: e.g. India [20] Thailand [21] and Taiwan [22]. Though many functional ability scales exist, they have not been validated for the elderly population in Sri Lanka. The purpose of this study was to derive norms for Activities of Daily Living (ADL) scale among the elderly living in care homes in the suburbs of Colombo, Sri Lanka and examine the usefulness of Bristol [23] and short form of Blessed ADL (Blessed-CERAD version) [24] scales to detect dementia in care homes.

## Methods

### Study Population

From a list of elderly homes maintained by the Department of Social Services [17], 14 elder's homes from Western province of Sri Lanka were randomly selected according to the geographical distribution. For example, we selected 2 homes from the Colombo South to represent the elder's homes in Colombo South.

A total of 73 elderly people aged 55 years and above were interviewed for the study. Exclusion criteria were any of the following: blindness, deafness, physically disable (due to medical problems other than dementia) and being unable to communicate.

Ethical clearance was obtained from the Faculty of Medical Sciences, University of Sri Jayewardenepura, permission to carry out the study was obtained from the Director of each of the respective institutions and informed written consent was obtained from the participants/caregivers of the participant.

### ADL scales and administration

We chose the Bristol and short form of Blessed Activities of Daily Living (ADL) scales for this study. These scales were modified to suit the sociocultural needs of Sri Lanka (taking into consideration of common activities carried out by the elderly population living in care homes in Sri Lanka), since certain tasks are not performed by the elders living in elders homes those tasks were removed from the scale. For example: preparing food and drink, hygiene (washing cloths), gardening/house work, shopping, finances and transport. Instead the following two items were included in the scale: "taking medications on their own" and "ability to remember important festivals". Furthermore, the following two items were modified: ability to use the cheque book item was modified to "handling the money on their own" and the ability to answer telephone call item was modified to "ability to relay messages" and then modified scale was translated into Sinhalese language. A group of different experts back translated the scales to English. The modified instruments were pilot tested on a random sample of 30 elder people before being used in the present study.

The original Bristol and short form of Blessed scales consist of 20 and 11 items respectively. However, the modified Bristol and Blessed scales (Additional file 1) consist of 14 and 13 items respectively and the total score of modified Bristol scale ranges from 0 (independent on their activities) to 42 (dependent on their activities) while the total score of modified Blessed scale ranges from 0 (independent) to19 (dependent).

### Determination of cognitive status

All subjects were assessed by a Consultant Psychiatrist with special interest in Geriatric Psychiatry who diagnosed dementia according to the Neurological Adaptation of the 10^th ^edition of the International Classification of Disease (ICD-10NA). Subjects who were diagnosed as demented were further evaluated by Psychiatrist and their severity of dementia was rated using the Clinical Dementia Rating (CDR) scale [25]. Finally, both the modified ADL scales were administered by a research assistant, blind to the subject's cognitive status to the caregivers of each subject to obtain scores on the modified Bristol and Blessed scales. The diagnostic performances of the modified Bristol and Blessed scales were then compared against the clinical diagnosis of Psychiatrist's assessment which was considered the 'gold standard'.

In this study, severity of dementia was categorized using the Sinhalese version of Clinical Dementia Rating (CDR) scale which was found to be a sensitive and culturally adapted screening tool for dementia in Sri Lanka [26]. CDR ratings are 0 for healthy people, 0.5 for questionable dementia and 1, 2, 3 for mild, moderate and severe dementia, respectively. However, given the relatively small number of subjects in our study, the CDR scores of moderate and severe dementia (CDR values of 2 and 3 respectively) were grouped together to enable a more valid comparison. Since the subjects with CDR scores (CDR values of 0.5) of questionable dementia cannot be grouped into either non demented or mild demented category, they were excluded from the study (3 subjects).

### Statistical Analysis

Receiver operating characteristic (ROC) technique was used to compare the diagnostic performance of the modified Bristol and Blessed scales. The optimal cutoff scores for each scale as well as the sensitivity and specificity of both the scales were determined. All analyses were carried out using the SPSS Version 13.0 Software (2005, Chicago) [27].

## Results

### Demographics

The final cohort consisted of 31 demented elderly subjects and 39 controls. The age and sex distribution of the study sample are shown in Table 1. There was a female preponderance (n = 52, 74.3%) in the sample and the majority of the subjects (n = 33, 47.1%) were over 75 years of age. Summaries of the scores on the ADL scales (Bristol and Blessed), obtained from the non demented, mild demented and moderate - severe demented groups are shown in Table 2.

**Table 1:** Demographic characteristics

	Non Demented	Mild demented	Mod -Sev demented	Total
	(n = 39)	(n = 17)	(n = 14)	(n = 70)
	n (%)	n (%)	n (%)	n (%)
**Age (years)**				
55-64	5 (12.8)	4 (23.5)	1 (7.14)	10 (14.3)
65-74	19 (48.7)	4 (23.5)	4 (28.6)	27 (38.6)
≥75	15 (38.5)	9 (52.9)	9 (64.3)	33 (47.1)
				
**Gender**				
Male	15(38.5)	1 (5.9)	2 (14.3)	18 (25.7)
Female	24(61.5)	16 (94.1)	12 (85.7)	52 (74.3)
				
**Education**				
≤ 5 years	23 (59)	14 (82.4)	12 (85.7)	49 (70)
6-10 years	9 (23.1)	1 (5.9)	1 (7.1)	11(15.7)
>10 years	7 (17.9)	2 (11.8)	1 (7.1)	10 (14.3)
				
**Median age**	72 (57-93)	75 (57-93)	76 (60-94)	74 (57-94)

n = total number of subjects

**Table 2:** Descriptive scores of Bristol and Blessed scales and mean values of CDR score

	Non Demented	Mild Dementia	Mod -Sev Dementia
	n = 39	n = 17	n = 14
**Bristol scores**			
Mean (Std.Err)	4.46 (0.55)	10.59 (0.9)	22 (2.23)
Median (range)	4 (0-16)	10 (3-17)	21.5 (12-42)
**Blessed scores**			
Mean (Std.Err)	1.85 (0.27)	4.38 (0.56)	11.5 (0.97)
Median (range)	1.5 (0-6)	5 (1-9)	11 (5.5-19)
**CDR scores**			
Mean	-	1	2.28

### Diagnostic performance of the scales

**Figure 1:**
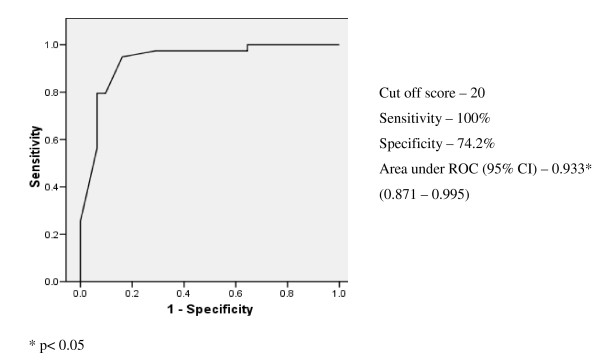
**Bristol - Non demented (n = 39) vs demented (n = 31) ROC curve**.

**Figure 2:**
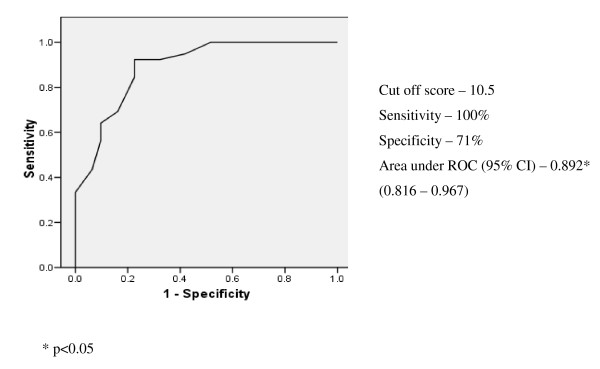
**Blessed - Non demented (n = 39) vs demented (n = 31) ROC curve**.

ROC analysis was carried out on the ADL scale scores obtained from the non demented and demented (mild demented and moderate - severe demented were grouped together) groups and the sensitivity, specificity and cut off values of both the scales were determined (Figures 1 &2). The optimal cut off score of the modified Bristol scale was 20 in differentiating non demented from demented with a sensitivity of 100%, specificity of 74.2% and the area under the curve 0.933(95% CI: 0.871-0.995) while the optimal cut off score of the modified Blessed scale was 10.5 in differentiating non demented from demented with a sensitivity of 100%, specificity of 71% and the area under the curve 0.892 (95% CI: 0.816-0.967).

## Discussion

Our study demonstrates that at their optimal cut off scores both the modified Bristol (sensitivity 100%, specificity 74.2%) and Blessed (sensitivity 100%, specificity 71%) ADL scales had a high sensitivity and specificity for detecting dementia and thus can be used to screen dementia in elderly Sri Lankan population living in care homes. The area under the ROC curve (AUC) is a reasonable summary of the overall diagnostic accuracy of the test. An area of 0.9-1 represents an excellent test and an area of 0.8-0.9 represents a good test [28] and from that perspective the modified Bristol scale (area under ROC: 0.933) is a better instrument than the modified Blessed scale (area under ROC: 0.892). Though the modified Bristol scale had a better ROC area under the curves (Figure 1) which is a valuable feature for a screening instrument the modified Blessed scale has the advantages of being simple to administer and interpret.

In developing countries, different ADL scales have been developed and used with a wide range of sensitivities and specificities [20-22,29]. The Everyday Abilities Scale for India [EASI] is an 11 item ADL scale developed for the illiterate elderly population in the Ballabgarh rural area in North India [20]. This study was conducted on people over 55 years of age and suggested an operational cut off point of 3, yielding a sensitivity of 62.5% and specificity of 89.7% [20]. The Chula Mental Test (CMT) was developed to screen for functional impairment in the Thai elderly population. The CMT at its optimal threshold had the best combination of sensitivity (100%) and specificity (90%) for detection of dementia [29], which is consonant to the findings of our study. Validation of the short form of Blessed scale has also been done in the Taiwanese population and suggested a cut-off score which differ with age and literacy of the study population [22].

Our study has several limitations, one of which is its relatively small sample size, especially when the analysis was conducted within the sub groups of mild and moderate-severe dementia. Due to the relatively small sample size, optimal cut off scores for mild and moderate - severe dementia were not defined in this study. Therefore, the accuracy of our findings would thus need to be replicated in larger studies. Second limitation was that both the scales were administered to the caregivers, because we thought that the responses from patients with dementia may not be accurate and thus decided upon caregiver administration of the scales. Since in these Elder's Homes, all the members (average of 30 members per home) are looked after by 2 - 3 care givers and we assumed that the information obtained from these care givers about each member is reliable. However it has been showed that the informant's personal characteristics contribute to contrasting results between the informant's reports and direct assessment of activities of daily living in patients affected by very mild dementia [30]. Furthermore, care givers from each elders home already knew about the cognitive status of the elderly person, this may have an impact on their assessment with the ADL scales. But the informant based scale has the advantage of allowing patients to be evaluated over the full range of their abilities despite communication difficulties and it allows evaluation of change over time [30]. Third limitation was both the scales were administered by the same interviewer this may have influenced the rating of the second scale. However, we decided upon single interviewer administration to avoid the difference in scoring system (to avoid the observer variation between two people). Another limitation was exclusion of people with physical disability (due to medical problems other than dementia). Even though, the screening instruments can be used to identify dementia in the elderly, including those with physical disabilities, questions included in both the ADL scales (Bristol and Blessed) can be influenced by the physical disability of a person (for example, bathing, toileting, mobility, etc), hence we decided to exclude the people with physical disability in this study. Finally, the Sinhalese versions of the Bristol and Blessed ADL scales used in this study were substantially modified for the elderly Sri Lankan population living in care homes, because the original versions clearly both culturally and linguistically inappropriate for the population in Sri Lanka. Therefore, our instruments (both Bristol and Blessed) are only specific for the elderly Sri Lankan population living in care homes. Furthermore, due to the relatively small sample size, age and literacy specified cut offs were not suggested for our study population (as done in Taiwan study [22]). However, with further research, the modified Bristol and Blessed scale could be applied in different settings to compare and age and literacy specified cutoffs could be suggested if needed.

In conclusion, both the modified Bristol and Blessed scales were equally effective in screening for dementia in the elderly Sri Lankan population living in care homes with due consideration of advantages and disadvantages of each.

## Competing interests

The authors declare that they have no competing interests.

## Authors' contributions

US - Data collection and drafted the manuscript; KM - Clinical Psychiatrist; SS - Statistical analysis; SS - Designed the study and coordination; KS - Designed the study and coordination; RDS - conceived of the study, and participated in its designed and coordination. All the authors have read and approved the final manuscript

## Supplementary Material

Additional file 1 **Modified Bristol and Blessed ADL scales**. questionnaires of modified Bristol and Blessed ADL scales. Click here for file

## References

[B1] Folstein MF Folstein SE McHugh PR 'Mini Mental State': a practical method for grading the cognitive state of patients for the clinician J Psychiatr Res 1975 12 189 198 10.1016/0022-3956(75)90026-6 1202204

[B2] O'Connor DW Pollit PA Hyde JB The reliability and validity of the Mini-Mental State in a British community survey J Psychiatr Res 1989 23 87 96 10.1016/0022-3956(89)90021-6 2666647

[B3] Zhang M Katzman R Salmon D The prevalence of dementia and Alzheimer's disease in Shanghai, China: impact of age, gender and education Ann Neurol 1990 27 428 37 10.1002/ana.410270412 2353798

[B4] Fratiglioni L Grut M Forsell Y Prevalence of Alzheimer's disease and other dementias in an elderly urban population: relationship with age, sex and education Neurology 1991 41 1886 92 1745343 10.1212/wnl.41.12.1886

[B5] Ylikoski R Erkinjuntti T Sulkava R Correction for age, education and other demographic variables in the use of the Mini-Mental State Examination in Finland Acta Neurol Scand 1992 85 391 6 10.1111/j.1600-0404.1992.tb06034.x 1642109

[B6] De Silva R Disanayaka S De Zoysa N Norms for the mini-mental state examination from an elderly Sri Lankan sample Int J Geriatr Psychiatry 2009 24 666 670 10.1002/gps.2168 19132690

[B7] Juva K Makela M Erkinjuntti T Functional assessment scales in detecting dementia Age and ageing 1997 26 393 400 10.1093/ageing/26.5.393 9351484

[B8] Stern Y Andrews H Pittman J Diagnosis of dementia in a heterogeneous population. Development of a neuropsychological paradigm-based diagnosis of dementia and quantified correction for the effect of education Arch Neurol 1992 49 453 60 1580806 10.1001/archneur.1992.00530290035009

[B9] Barberger-Gateau P Commenges D Gagnon M Instrumental activities of daily living as a screening tool for cognitive impairment and dementia in elderly community dwellers J Am Geriatr Soc 1992 40 1126 1134 10.1111/j.1532-5415.1992.tb01802.x 1401698

[B10] Human development report, 2009 http://hdrstats.undp.org/en/countries/country_fact_sheets/cty_fs_LKA.html

[B11] Help Age India Aging Scenario Help Age India http://www.helpageindia.org 1-12-2003, Help Age India. 1-12-2006

[B12] Muthane UB Ragothaman M Gururaj G Epidemiology of Parkinson's disease and movement disorders in India: problems and possibilities J Assoc Physicians India 2007 55 719 724 18173026

[B13] World Bank 2006 Sri Lanka Aging Survey - SLAS, the survey based on a representative sample of Sri Lankan old people) http://siteresources.worldbank.org/INTSRILANKA/Resources/LKAgingExSum.pdf

[B14] Department of census & statistics, Sri Lanka, 2001 http://www.statistics.gov.lk/page.asp?page=Population and Housing

[B15] Annual health bulletin 2006 Ministry of Health Sri Lanka

[B16] De Silva I How serious is aging in Sri Lanka and what can be done about it? Asia Pac Popul 1994 9 19 36 12288068

[B17] Statistics 2006 Department of Social Services. Sri Lanka

[B18] Bronnick K Ehrt U Emre M De Deyn PP Wesnes K Tekin S Aarsland D Attentional defecits affect activities of daily living in dementia - associated with Parkinson's disease Journal of Neurology, Neurosurgery & Psychiatry 2006 77 1136 1142 10.1136/jnnp.2006.093146 PMC2077544 16801351

[B19] Avlund K Kreiner S Schultz-Larsen K Functional ability scales for elderly: A validation study European Journal of Public Health 1996 6 35 42 10.1093/eurpub/6.1.35

[B20] Fillenbaum GG Chandra V Ganguli M Development of an activities of daily living scale to screen for dementia in an illiterate rural older population in India Age and Ageing 1999 28 161 168 10.1093/ageing/28.2.161 10350413

[B21] Jitapunkul S Kamolratanakul P Ebrahim S The meaning of Activities of Daily Living in a Thai elderly population: Development of a new index Age and aging 1994 23 97 101 10.1093/ageing/23.2.97 8023736

[B22] Yang Y Lai C Lin R Tai C Liu C Cut-off values of Blessed dementia rating scale and its clinical application in elderly Taiwanese Kaohsiung J Med Sci 2006 22 377 84 10.1016/S1607-551X(09)70326-2 16911919 PMC11917648

[B23] Bucks RS Ashworth DL Wilcock GK Siegfried K Assessment of Activities of Daily Living in Dementia: Development of the Bristol Activities of Daily Living Scale Age and Aging 1996 25 113 120 10.1093/ageing/25.2.113 8670538

[B24] Morris JC Heyman A Mohs RC The Consortium to Establish a Registry for Alzheimer's disease (CERAD): Part I. Clinical and neuropsychological assessment of Alzheimer's disease Neurology 1989 39 1159 65 2771064 10.1212/wnl.39.9.1159

[B25] Hughes CP Berg L Danziger WL A new clinical scale for the staging of dementia Br J Psychiatry 1982 140 566 572 10.1192/bjp.140.6.566 7104545

[B26] Kathriarachchi ST Sivayogan S Jayaratna SD Dharmasena SR Comparison of three instruments used in the assessment of dementia in Sri Lanka Indian J Psychiatry 2005 47 109 112 10.4103/0019-5545.55957 20711293 PMC2918294

[B27] SPSS PC Version, 13.0, 2005 SPSS Inc: Chicago, IL, USA

[B28] Akobeng AK Understanding diagnostic tests 3: receiver operating characteristic curves (Review article) Acta Paediatrica 2007 96 644 647 10.1111/j.1651-2227.2006.00178.x 17376185

[B29] Jitapunkul S Lailert C Worakul P Srikiatkhachorn A Ebrahim S CHULA mental test: A screening test for elderly people in less developed countries Int J Geriatr Psychiatry 1995 11 715 720 10.1002/(SICI)1099-1166(199608)11:8<715::AID-GPS374>3.0.CO;2-Q

[B30] Zanetti O Geroldi C Frisoni GB Consulting results between caregiver's report and direct assessments of activities of daily living in patients affected by mild and very mild dementia: The contribution of the caregiver's personal characteristics J Am Geriatr Assoc 1999 47 196 202 10.1111/j.1532-5415.1999.tb04578.x 9988291

