# Supplementary material for: Validation of a functional screening instrument for dementia in an elderly sri lankan population: comparison of modified bristol and blessed activities of daily living scales
Source: BMC Res Notes. 2010 Oct 26;3:268. doi: 10.1186/1756-0500-3-268 (PMC2987868; doi:10.1186/1756-0500-3-268)
Supplement: Additional file 1 — Modified Bristol and Blessed ADL scales. questionnaires of modified Bristol and Blessed ADL scales. [file 1756-0500-3-268-S1.DOC]

**Appendix**

**Modified BRISTOL ADL Scale** **Points**

1. Eating

a. Normal 0

b. Independent but slow or some pills 1

c. Needs some help, avoid some foods, spills often 2

d. Must be fed most foods 3

2. Drinking

a. Drinks appropriately 0

b. Drinks appropriately with aids, straw, etc 1

c. Doest not drink appropriately even with aids but attempts to 2

d. Has to have drinks administered (fed) 3

3. Taking Medication

a. Remembers without help 0

b. Remembers if left in special places 1

c. Tries but forgets frequently if not reminded 2

d. Medication must be given by others 3

4. Dressing

a. Selects appropriate clothing and dress itself 0

b. Puts cloths in wrong order 1

c. Unable to dress self, but moves limbs to assist 2

d. Unable to assist and requires total dressing 3

5. Teeth

a. Cleans regularly & independently 0

b. Cleans teeth if given appropriate items 1

c. Requires some assistance 2

d. Full assistance given 3

6. Bath

a. Bath regularly and independently 0

b. Needs bath to drawn/ shower turned on but washes

independently 1

c. Needs supervision & prompting to wash 2

d. Totally dependent 3

7. Toilet

a. Uses toilet appropriately when required 0

b. Needs to be taken to the toilet & given assistance 1

c. Incontinent of urine or faces 2

d. Incontinent of urine & faces 3

8. Mobility

a. Walks independently 0

b. Walks with assistance 1

c. Use aids to mobilize, i.e. sticks, etc 2

d. Unable to walk 3

9. Orientation - Time

a. Fully orientated to time/day/date, etc 0

b. Unaware of time/day but seems unconcerned 1

c. Repeatedly asks the time/day/date 2

d. Mixes up night & day 3

10. Orientation – Space

a. Fully orientated to surroundings 0

b. Orientated to familiar surroundings only 1

c. Gets lost in home, needs reminding where bathroom is, etc 2

d. Does not recognize home as own & attempts to leave 3

11. Hobbies/TV/Prayers/Group activities

a. Same interest as before 0

b. Less interest but still does on occasion 1

c. Reluctant to join in, very slow, needs coaxing 2

d. No longer able to or willing to join in 3

12. Communication

a. Able to hold appropriate conversation 0

b. Shows understanding & attempts to respond verbally with

gestures 1

c. Can make self understood but difficulty understanding others 2

d. Does not respond to or communicate with others 3

13. Telephone/ Relay messages

a. Uses telephone/ relay messages appropriately 0

b. Occasionally use telephone/ relay messages 1

c. Answers telephone but does not make calls/ relay messages,

commonly forgetting some of the messages 2

d. Unable to use telephone/ relay messages at all 3

14. Remember important festivals

a. Able to remember important festivals 0

b. Able to remember most of the festivals 1

c. Tries but forget frequently if not reminded 2

d. Unable to remember festivals 3

**Modified BLESSED ADL Scale**

**Changes in performance of everyday activities Points**

1. Inability to participate in group activities/ prayers/ watching TV 1 ½ 0

2. Inability to cope with small sums of money 1 ½ 0

3. Inability to take medications on his/her own 1 ½ 0

4. Inability to find way about indoors 1 ½ 0

5. Inability to travel to places out of walking distances on his/her own 1 ½ 0

6. Inability to interpret surroundings (e.g to recognize whether in hospital or at home) 1 ½ 0

7. Inability to recall recent events (e.g visits of relatives/ festivals) 1 ½ 0

8. Inability to communicate 1 ½ 0

9. Inability to orientate time 1 ½ 0

10. Inability to use telephone/ answers the telephone/ relay messages 1 ½ 0

11. Eating

a. Cleanly with proper utensils 0

b. Independent, but slow or some spills 1

c. Needs some help, spills often 2

d. Has to be fed 3

12. Dressing

a. Unaided 0

b. Occasionally misplaced buttons, etc 1

c. Wrong sequence, commonly forgetting items 2

d. Unable to dress 3

13. Continence

a. Complete sphincter control 0

b. Occasionally wets bed 1

c. Frequently wets bed 2

d. Doubly incontinent 3

**Scoring system for first 10 items**

0 – None

0.5 – Some

1 – Severe
